# Signal processing for slug flow analysis: MATLAB algorithm

**DOI:** 10.1016/j.mex.2021.101546

**Published:** 2021-10-11

**Authors:** Gabriel Soto-Cortes, Eduardo Pereyra, Cem Sarica, Carlos Torres, Auzan Soedarmo

**Affiliations:** a Autonomous Metropolitan University Campus Lerma, Mexico, Mexico; b McDougall School of Petroleum Engineering, The University of Tulsa, Tulsa, OK, USA; c School of Mechanical Engineering, University of Los Andes, Merida, Venezuela; d Schlumberger Norway Technology Center, Asker, Norway

**Keywords:** Holdup, Gas-liquid flow, Slug characterization, Slug frequency, Slug length

## Abstract

This paper presents an easy-to-use MATLAB© program to characterize slug flow, one of the most observed gas-liquid flow patterns in pipes. “BSignalProcessing2020.m” based on the study reported in [1] is a flexible, expandable, and adaptable statistical algorithm used to calculate the film and slug cut threshold values, the disregard cut value to group slug pulses, and the disregard cut value to remove slug pulses, which are required to determine the slug characteristics. The code is provided for unlimited and unrestricted use.

• The statistical algorithm does not depend on any subjective criteria.

• The methodology is illustrated using voltage time-series, but it is applicable without changes to instantaneous liquid holdup time-series.

## Graphical abstract

**Figure:**
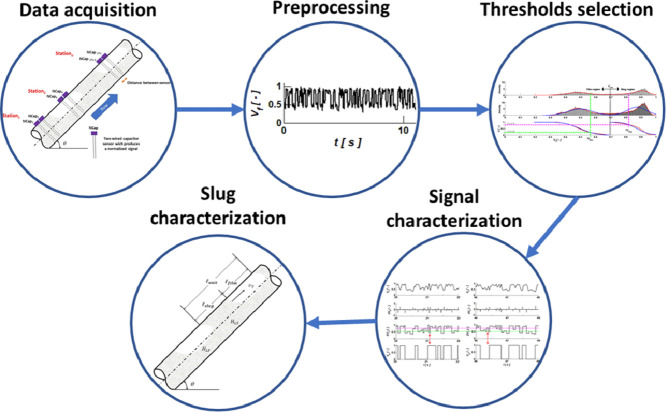

Specifications table

**Table:** 

Subject Area:	Engineering
More specific subject area:	*Liquid – gas flow in piplines.*
Method name:	*Signal processing for slug flow analysis via a voltage or instantaneous liquid holdup time-series.*
Name and reference of original method:	*G.E. Kouba, Horizontal slug flow modeling and metering, University of Tulsa, 1986. E.M. Al-Safran, An experimental and theoretical investigation of slug flow characteristics in the valley of a hilly-terrain pipeline, University of Tulsa, 2003.*
Resource availability:	*MATLAB© script with this paper: “BSignalProcessing2020.m” and its complementary data to run the examples are available here as supplementary material.*

## Introduction

The concept development of the signal processing methodology reported in [1] started in 2014 as a project of the Tulsa University Fluid Flow Projects (TUFFP) [2,3]. The method is inspired by the studies of Kouba [4] and Al-Safran [5]. Kouba [4] presented one of the first studies with a formal description of a signal processing algorithm based on a voltage time-series introducing the idea of one threshold cut value. Al-Safran [5] modified the methodology, including separate threshold values for the slug front and tail, to reduce the measurement uncertainty. Later, Brito [6] cautioned about the uncertainty propagated for an incorrect threshold value selection. Her observations showed the need to develop a universal signal processing algorithm, which would reduce or eliminate human subjectivity and would be implemented by the research community to reduce the variability of results during the characterization of the slug parameters. At the beginning (2014), some of the algorithms now included in *BsignalProcessing2020* - a MATLAB© script - were developed specifically for the TUFFP's three-phase experimental facility described in [7,8]. In subsequent years, the methodology was improved and tested using the data sets acquired during the experimental campaigns reported in [3,7]. The code or some parts of it were used for the data analysis described in [9,10].

## Objectives and general description

*BsignalProcessing2020* is a computer code based on MATLAB© (R2014). It is a set of several algorithms presented as a single function with the following objectives:

- Systematize and synthesize a methodology for signal processing analysis that does not require any subjective parameter to calculate the film and slug cut threshold values, the disregard cut value to group slug pulses, and the disregard cut value to remove slug pulses, which are necessary to determine the slug characteristics.
- Offer an open code to the scientific community, which is flexible, expandable, and easily adaptable to different experimental facilities.

**Fig. 1:**
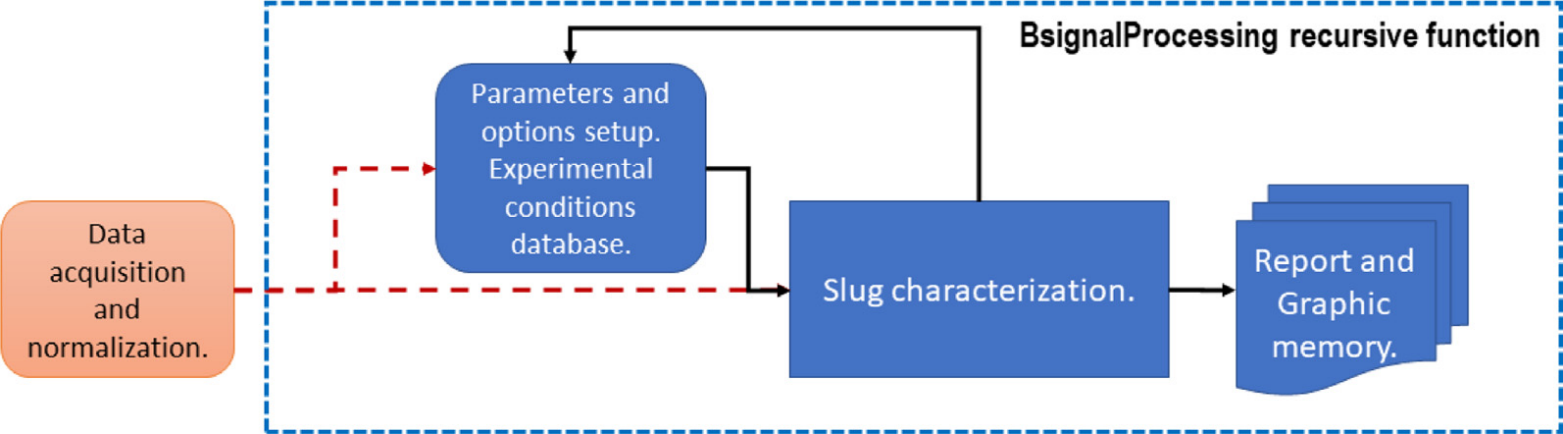
Schematic of BsignalProcessing's design structure.

*BsignalProcessing2020* has a simple user interface but with several run mode options. Fig. 1 shows a schematic of its structure.

1. Data acquisition and normalization: As the main input, *BsignalProcessing2020* uses voltage or instantaneous liquid holdup time-series, which come from a data acquisition station. The station consists of two sensors separated by a known distance. For a specific experimental condition, both signals are saved together in a mat file. *BsignalProcessing2020* can process at the same time data from one or several stations and one or several experimental conditions.
2. Parameters and options setup, and Experimental conditions database: A Microsoft Excel workbook (xls) is used as a data flow control interface. In a sheet of this workbook, the experimental conditions for each experiment are registered. An *experiment* is defined by seven variables: experiment trial, superficial liquid velocity, superficial gas velocity, temperature, pipe inclination angle, data acquiring time interval, and stations available. In different sheets, the run modes, input, and output parameters are specified.
3. Slug characterization: The analysis process is performed according to user specifications. The code displays information on the MATLAB© desktop while it is running to follow the process and help the user to take actions if necessary or if the user selected an interactive mode.
4. Graphic memory: For each experiment, a fig file is saved for its further analysis and edition.
5. For clarity and order, the analysis report is saved in the same xls file. For each experiment, the report includes several variables as a result of the slug analysis. They are related to translation velocity, slug length, and slug frequency.

### Main modules description

**Fig. 2:**
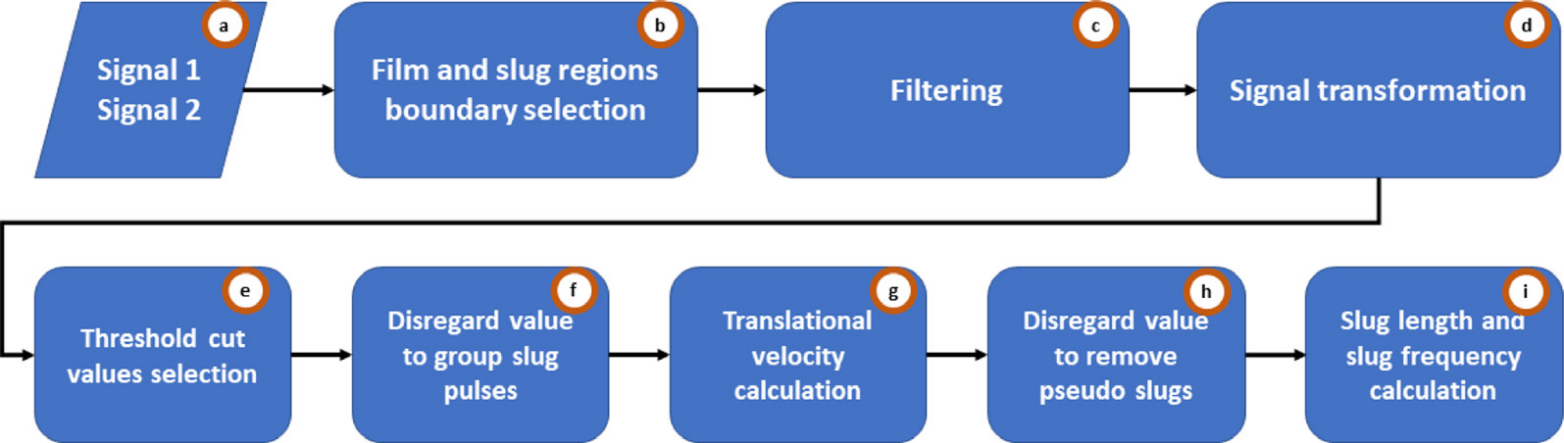
Schematic of BsignalProcessing's main modules.

BsignalProcessing's main modules are conceptually described in Fig. 2. The data flow could follow a linear trend as is illustrated, skip the filtering module, and run in different interactive modes (see Section 3).

A couple of normalized signals from an experimental station are necessary to execute the code (Fig. 2(a)). This is because the delay between them is a fundamental parameter for the translational velocity estimation and the slug lengths. Each signal is processed following the methodology described in [1]. The procedure starts fitting an empirical density function to the time series and identifies the boundary between slug and film regions (Fig. 2(b)). Raw normalized data can be used, or the high frequencies can be removed by a filtering process (Fig. 2(c)). This is to simplify the signal transformation process (Fig. 2(d)) from a quasi-continuous to a digital function. The probability distribution function fitted to the slug and film data allows identifying the threshold cut values, which are applied to the digital signal (Fig. 2(e)). Signals must be filtered again to group slug pulses that were separated by mistake (Fig. 2(f)) and remove traveling waves (pseudo slugs) (Fig. 2(g)). A cross-correlation process is used to estimate the translational velocity (Fig. 2(h)). Finally, the length and slug frequency are calculated (Fig. 2(i)).

## Operation

Appendix A includes the supplementary material used in the following subsections.

### Data preparation

**Fig. 3:**
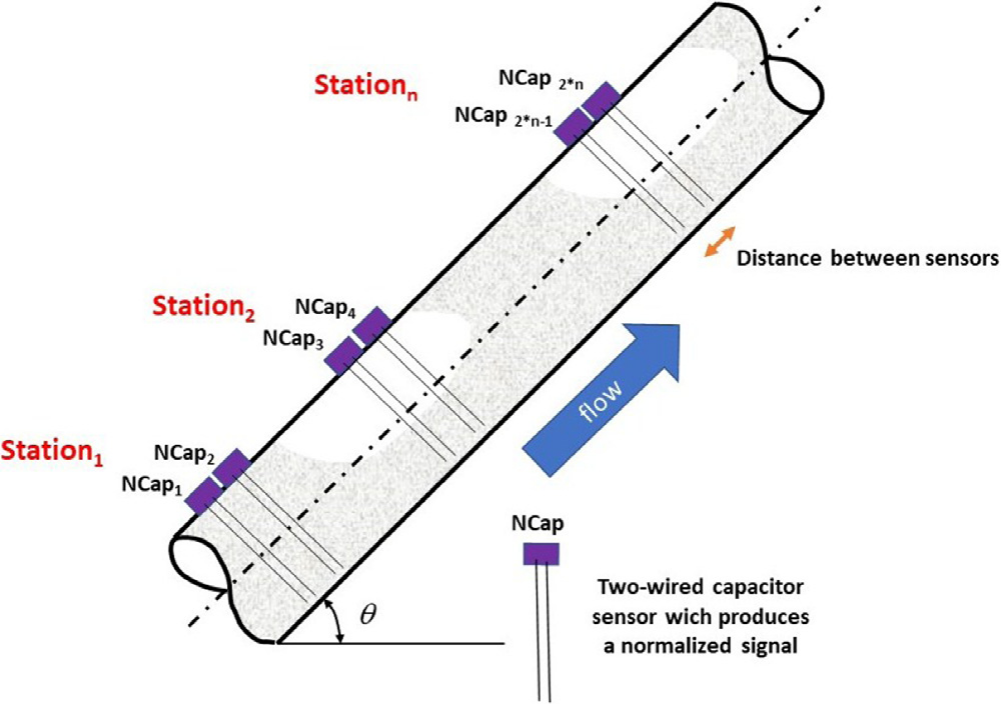
Schematic of an experimental facility.

As the main input, *BsignalProcessing2020*, uses voltage or instantaneous liquid holdup time-series, which come from a data acquisition station. The station consists of two sensors separated by a known distance. Every sensor is identified as *NCap_i_* where *i* is the sensor's number. Fig. 3 illustrates an experimental facility with *n* stations and 2*n* sensors.

**Fig. 4:**
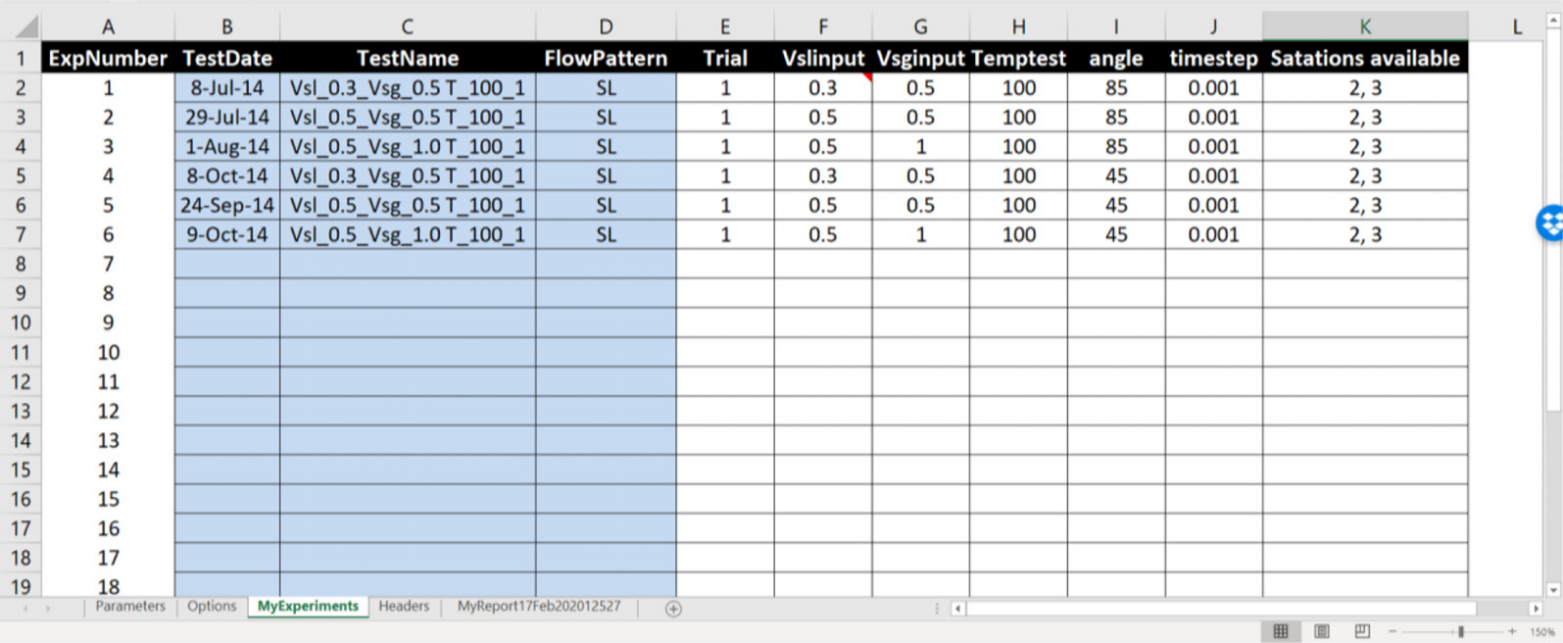
MyExperiments data sheet (data from [1]).

In *BsignalProcessing2020, NCap_i_* is a vector of normalized data acquired with a sampling frequency *f*_s_. The data are equally spaced in time by a time step ∆*t*. For a specific experimental condition (experiment *j*), both signals are saved together in a mat file with a name structure: Experiment_j_Station_i.mat. For example, Experiment_4_Station_2.mat must contain the *NCap*_7_ and *NCap*_8_ data vectors for the experiment named “4″. An xls workbook is used as a data flow control interface. In a sheet of this workbook (*MyExperiments*), the experimental conditions for each experiment are registered. An *experiment* is defined by seven variables: experiment trial, superficial liquid velocity, superficial gas velocity, temperature, pipe inclination angle, data acquiring time interval, and stations available. Fig. 4 shows a data set of 6 experiments. For each one, there are two working stations. It means that 6 × 2 = 12 mat files must be available for its analysis.

### Program execution parameters

**Fig. 5:**
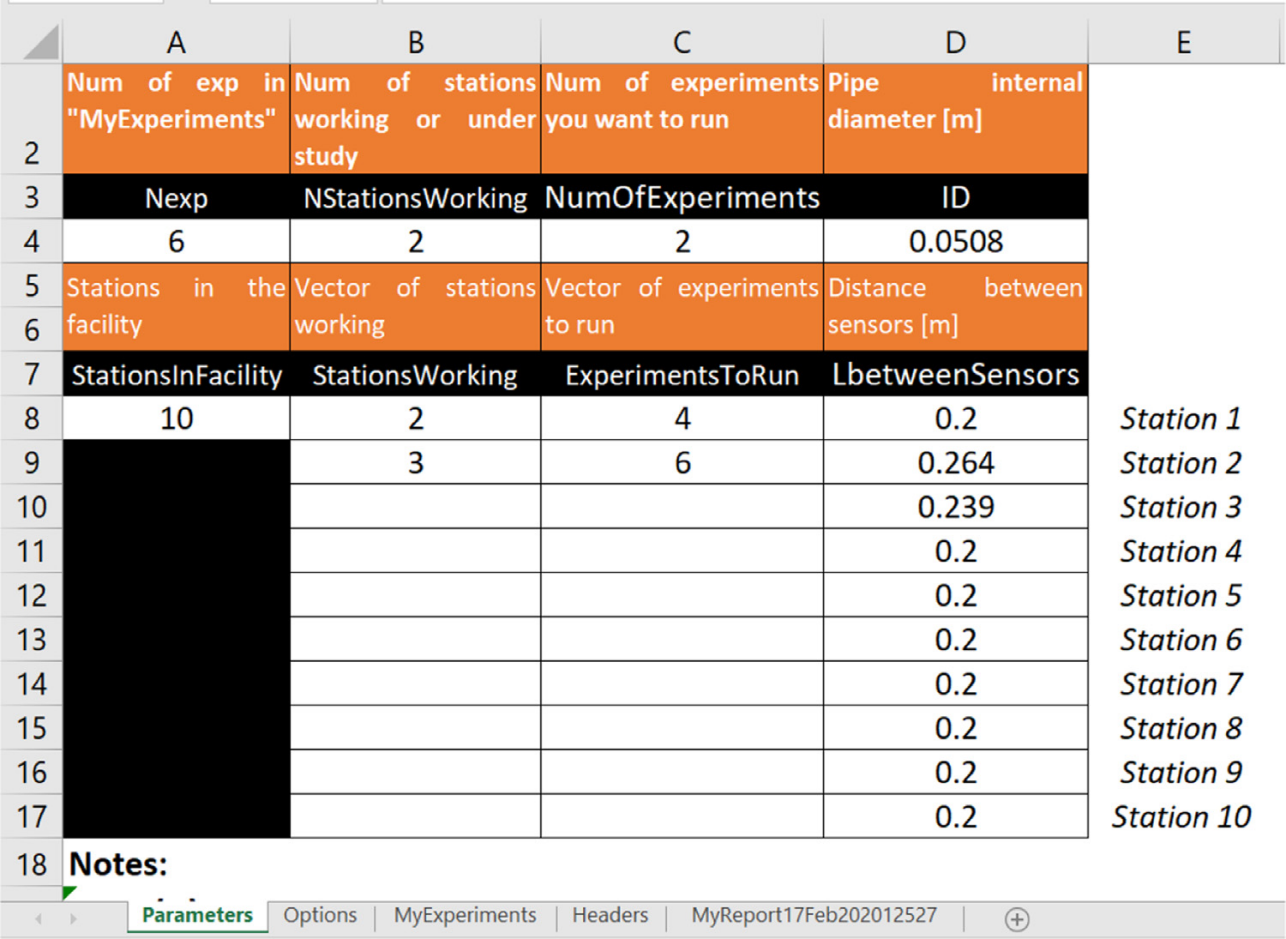
Parameters datasheet.

There are five variables and three arrays that work as input parameters (sheet *Parameters*). The variables are respectively: internal pipe diameter, the total number of experiments in *MyExperiments* sheet, the total number of stations in the facility, the number of stations, and the number of experiments. Arrays specify the distance between sensors for each station, station names, and experiment names. In the example shown in Fig. 5, the internal pipe diameter (ID) is 0.0508 m. The facility has ten stations, but only two of them, stations 2 and 3, will be analyzed. The distance between its sensors is 0.264 and 0.239 m, respectively. In this case, we will analyze only two sets of Fig. 4: experiments 4 and 6.

### Define the run mode

**Fig. 6:**
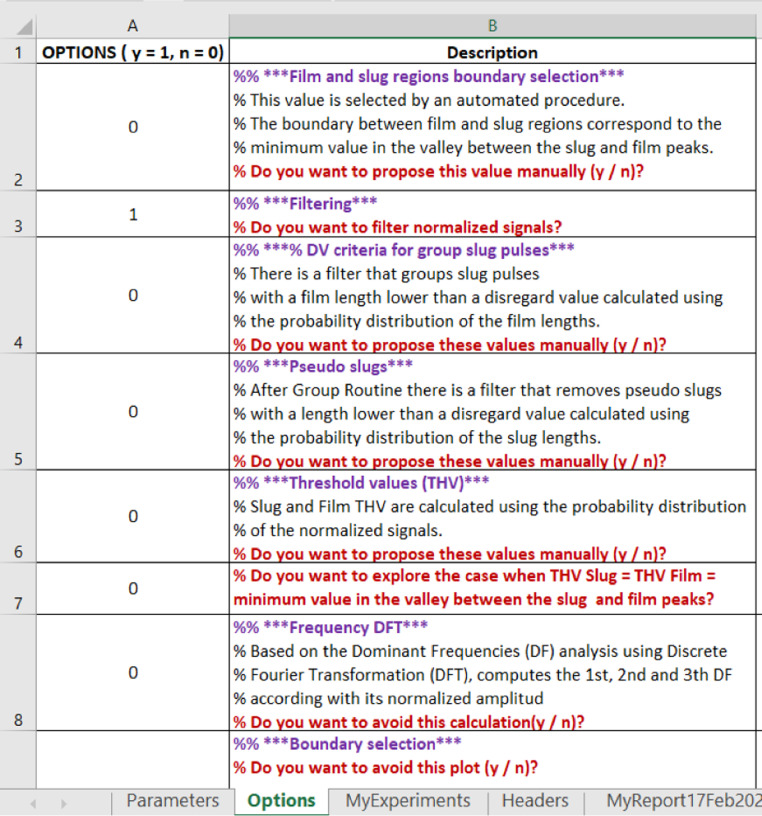
Options datasheet. Run modes.

The run modes are defined in the *Options* sheet (column A – rows 2–8, Fig. 6).

Run modes allow the user to explore several possibilities:

1. Film and slug regions boundary selection: This is performed by an automated procedure. The boundary between film and slug regions corresponds to the minimum value in the valley between the slug and film peaks (frequency histogram). With this option, the user can propose (or change) this value manually.
2. Filtering: There is a filtering procedure, which can be disabled by this option. Filtering removes the high frequencies – not corresponding to the slug pulse – considering them as noise.
3. The slug and film threshold cut values are estimated by an automated procedure using the probability distribution of the normalized signals. With this option, the user can propose (or change) this value manually.
4. Also, the user can explore the case when these film thresholds cut values are both equal to the minimum value in the valley between the slug and film peaks (a single threshold cut value).
5. There is a filter that groups slug pulses with a film length lower than a disregard value calculated using the probability distribution of the film lengths. With this option, the user can propose (or change) this value manually.
6. After the grouping routine, there is a filter that removes pseudo slugs with a length lower than a disregard value calculated using the probability distribution of the slug lengths. With this option, the user can propose (or change) this value manually.
7. Based on a power density spectrum analysis (PDF) using the discrete Fourier transformation (DFT), the code can compute the first, second and third dominant frequencies (DF) according to its normalized amplitude. The user can disable this calculation if it is not needed.

### Plot options

**Fig. 7:**
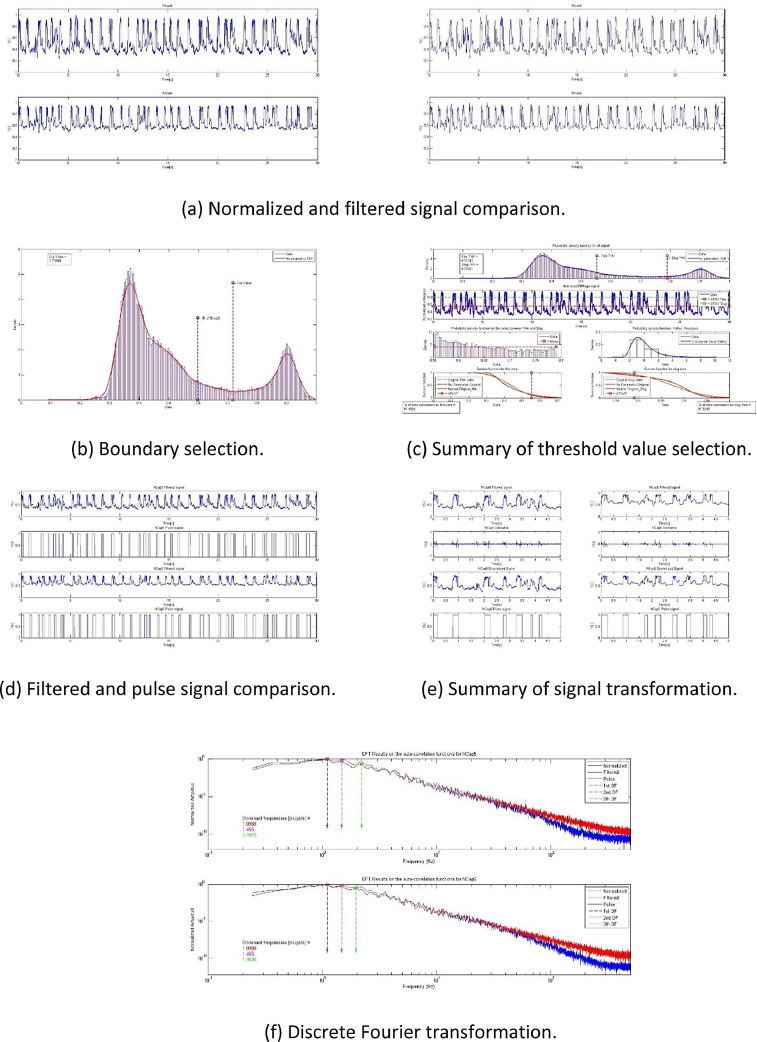
Optional graphs.

While BsignalProcessing2020 is running, several graphs are plotted to visualize the main results and help the user take actions if necessary. In the *Options* sheet, the user can disable some of these plots (Fig. 7)

### Data folder preparation

**Fig. 8:**
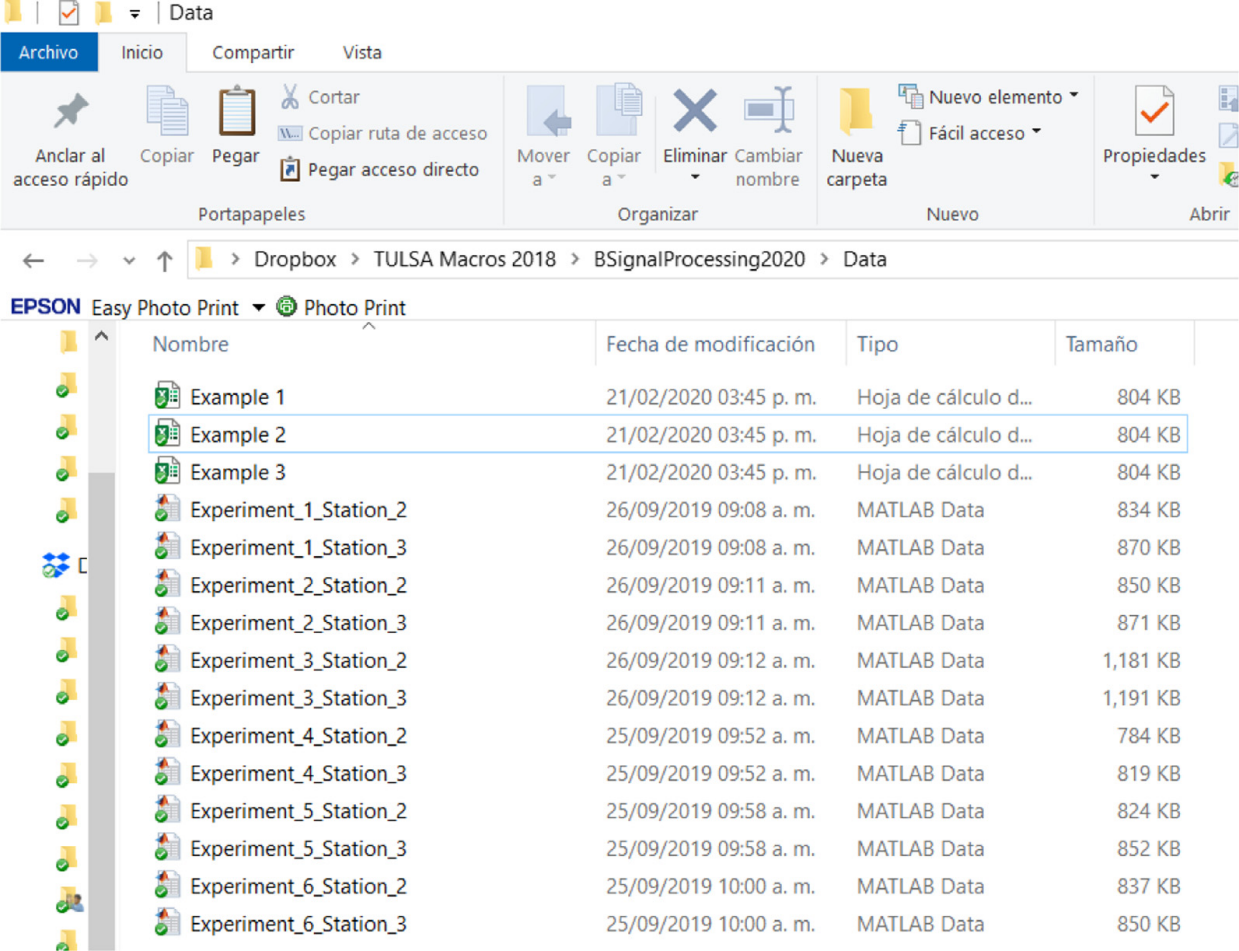
Example of a Data folder.

Place BSignalProcessing2020.m in the MATLAB© work folder or another convenient folder. Create a folder and put in your data files – Experiment_j_Station_i.mat – and the xls file or files. Fig. 8 illustrates an example of a Data folder based on the data set described in Fig. 4. In this example, there are three Excel files. They have the same information but different parameters and options. No more than one xls file is necessary except if the user wants to have the results in separate files.

### Outputs

Every time BSignalProcessing2020.m runs, two outputs will be generated: a written report, and a graph memory for each experiment and station.

#### Written report

For clarity and order, the analysis report is saved in the same xls file, in a sheet named *MyReport*. The tag includes date and hour to avoid duplicate it by mistake. For each experiment, the report includes several variables obtained from the slug analysis. *MyReport* has a simple format without headers. The user can copy and paste them from the *Headers* sheet included in the xls file. Variables in the report are grouped into 4 types: experimental conditions (Table 1), translational velocity - slug frequency (Table 2), slug length (Table 3), and threshold/options summary (Table 4). The experimental conditions come from *MyExperiments.* As mentioned in [1], translational velocity is calculated using the cross-correlation between the normalized signals (C21, Table 2). Also, using the cross-correlation of the digital signals, the front, and rear vectors, the code calculates the body, front and rear velocities (C17-C19, Table 2). The slug frequency for each signal (Fs, C25, and C37, Table 2) are calculated using the number of slug pulses counted by the code and the total test time. As mentioned in [1], a good estimation of the slug frequency is given by the frequency corresponding to the highest normalized magnitude obtained by the power density spectrum technic (PDS 1: C28 and C40, Magnitude of PDS 1: C31 and C43, Table 2). As complementary information, the second and third PDS frequencies and magnitudes are reported. The maximum, minimum, and average slug and film lengths (L_S_ and L_F_) are reported as the ratios L_S_ /ID and L_F_ /ID, including its standard deviations (std) (Table 3). ID represents the internal pipe diameter.

**Table 1:** Experimental conditions report.

**Table 2:** Translational velocity and slug frequency report.

**Table 3:** Slug and film lengths report.

**Table 4:** Threshold values and options report.

The report summarizes the disregard values (DV) and the threshold values (Table 4). Specifically, the boundary between slug and film, the disregard values to group pulses, and remove pseudo slugs, the threshold values for film and slug. Also, the option run modes and the figure options are reported. These options are described in Sections 3 and 4 of this user's guide.

#### Graph memory

In the Data folder for each experiment and station a fig file will be created with the following structure:

Exp_2_SL_Vsl_0.5_Vsg_0.5_100_85_1NCap3NCap4.fig

This example refers to the experiment 2, *v_sl_*= 0.5 m/s, *v_sg_* = 0.5 m/s, test temperature 100°F, inclination angle θ = 85°, trial 1, sensors 3 and 4 (station 2). The number of plots in each file depends on the selected operation parameters.

## Examples

Based on the supplementary material presented in Appendix A, this section presents three examples to illustrate the operation of *BSignalProcessing2020*.

### Example 1-Non-interactive mode

**Fig. 9:**
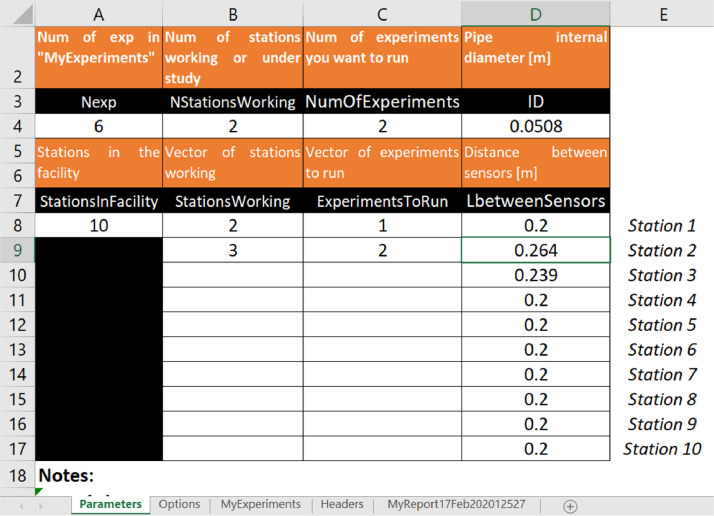
Parameters sheet for Example 1.

Consider the case in which we want to analyze the experiments 1 and 2 for the stations 2 and 3 (Example 1.xlsx) in a non-interactive mode (automated procedure), filter the normalized signals, and review all the optional graphs. In the *Options* sheet (column A rows 2 to 15), all the values are zero (*0 = “no”*) except the filter option (column A - row 3) that must be the value of one (*1 = “yes”*). On the other hand, the *Parameters* sheet must look like in Fig. 9.

**Fig. 10:**
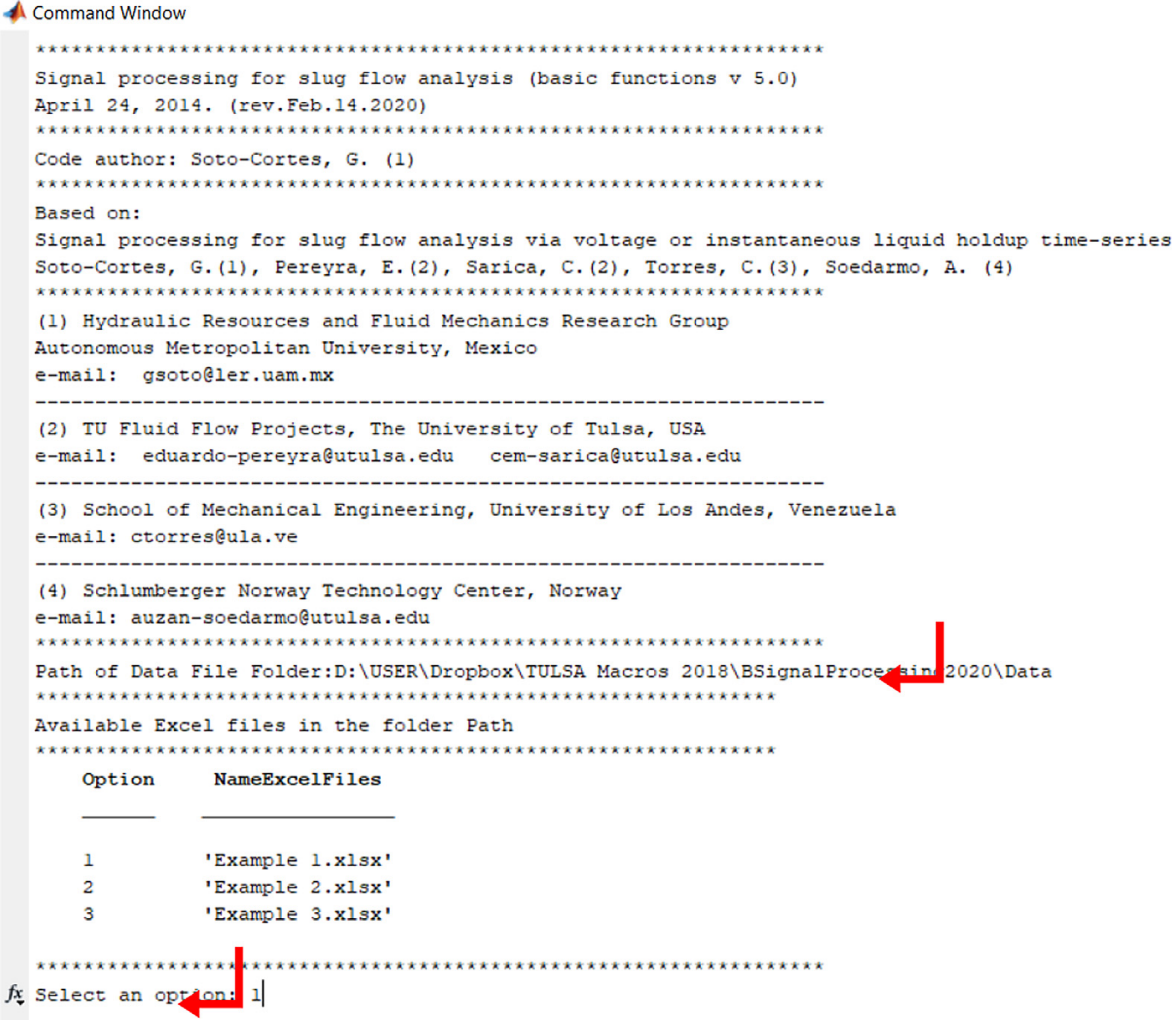
Path of the data file folder and Excel files available.

Make sure thatExample 1.xlsxis closed. Run BSignalProcessing2020.m. The code asks for the path of the data file folder. From the file explorer, copy and paste the path and press Enter. A list of the xls files existing in the folder will be displayed. Select the option 1 and press Enter (Fig. 10).

The analysis process is performed according to user specifications. The code displays information on the MATLAB© desktop while it is running, but the user can not interact with the software in this mode. In the data folder, a fig file is saved for each experiment and for each station (in this example, 2 × 2 = 4 files must be created). Moreover, in Example 1.xlsx file, a new sheet appears. Sheet's name starts with the legend MyReport followed by day, month, year, and hour. Text starts in Line 5. The user can copy headers from Headers sheet and paste them in MyReport (lines 1 to 4).

### Example 2-Interactive mode

**Fig. 11:**
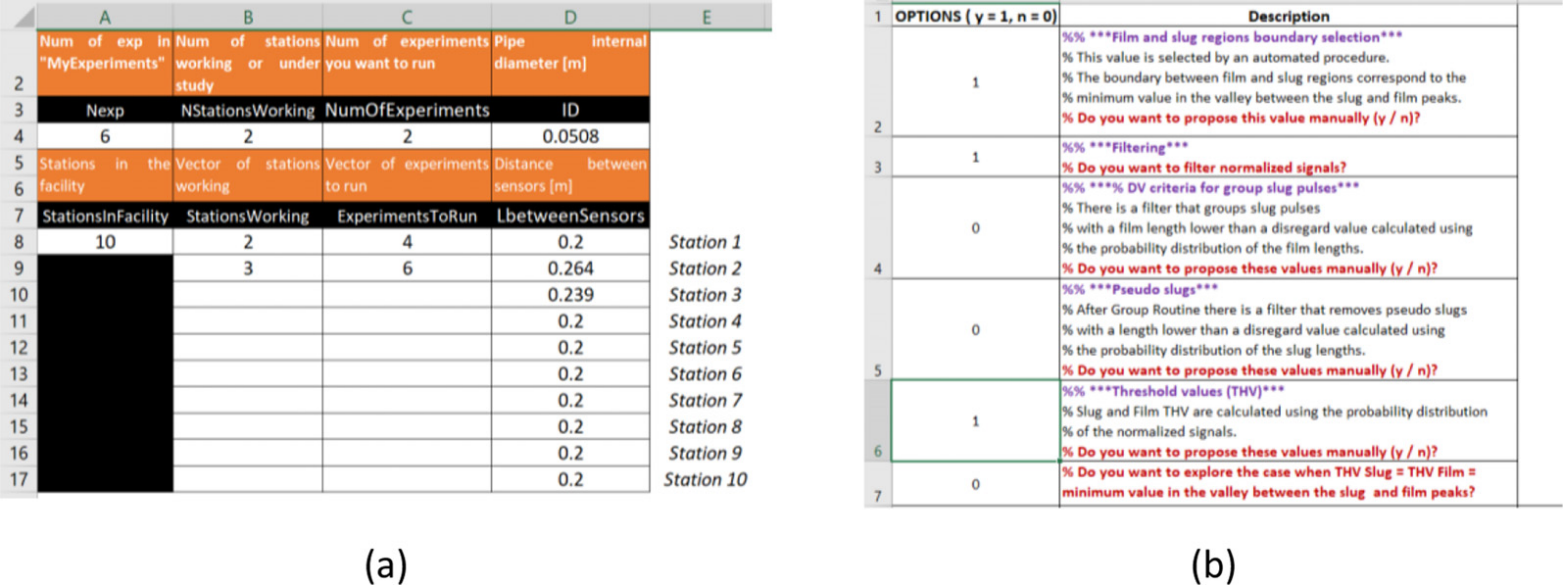
(a)Parameters, and (b)Options sheets for Example 2.

In this example, we want to analyze the experiments 4 and 6 for the stations 2 and 3 (Example 2.xlsx, the *Parameters* sheet must look like in Fig. 11(a)). We also want to introduce the film-slug boundary and the threshold values manually (interactive mode), filter the normalized signals, and review all the optional graphs. Then in the *Options* sheet, cells A2, A3, and A6 are equal to 1 (Fig. 11(b)).

**Fig. 12:**
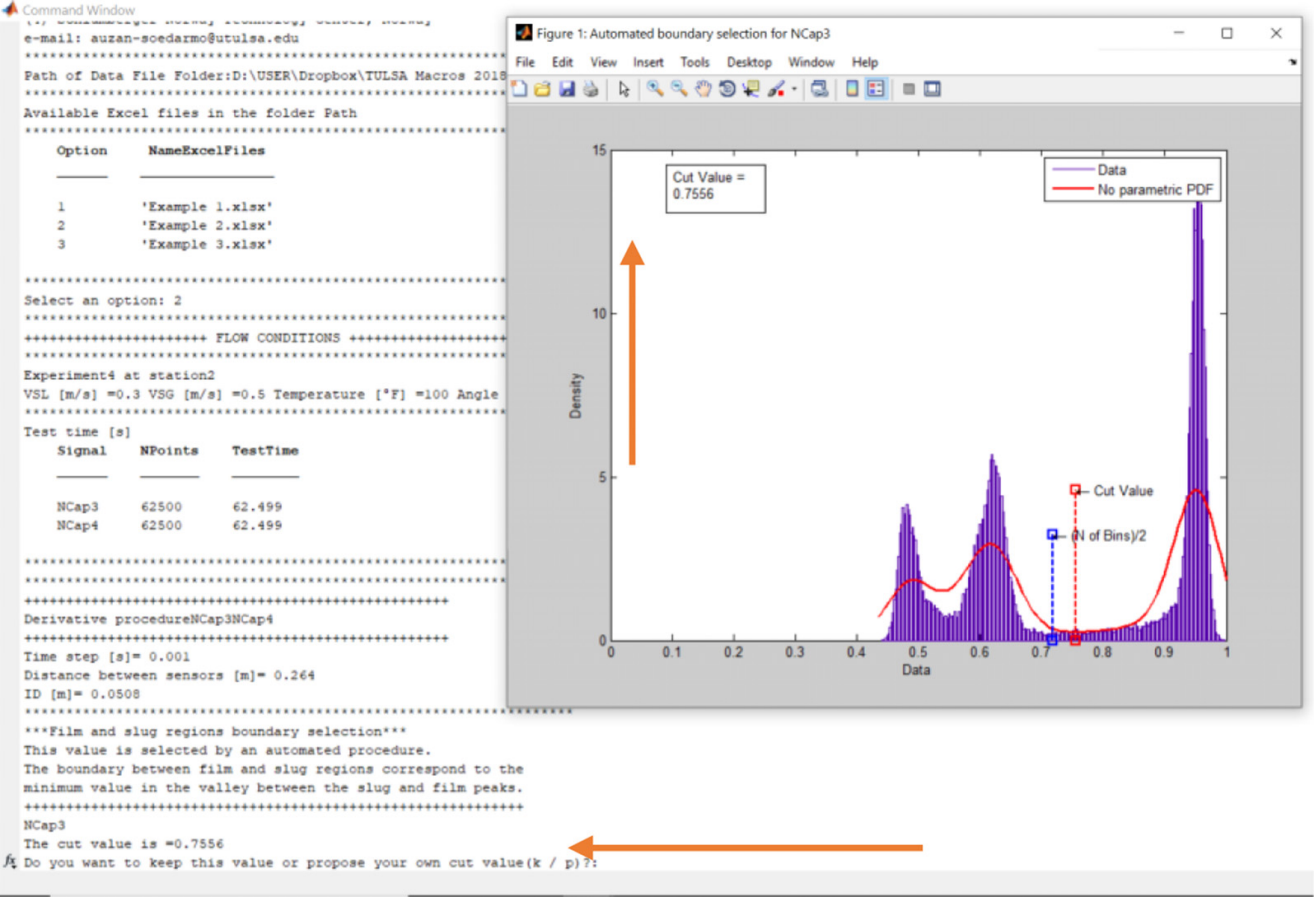
Selecting custom cut value.

Make sure thatExample 2.xlsxis closed. Run BSignalProcessing2020.m. The code asks for the path of the data file folder. From the file explorer, copy and paste the path and press Enter. A list of the xls files existing in the folder will be displayed. Select Option 2 and press Enter. The code asks for film-slug boundary value and waits for a response (Fig. 12): Do you want to keep this value or propose your own cut value (k / p)? If the user wants to keep the value (0.7556), just clicks Enter. In this example, we wrote p + Enter and proposed the value 0.8. The user must answer this question for each capacitance sensor, in each station, and each experiment.

**Fig. 13:**
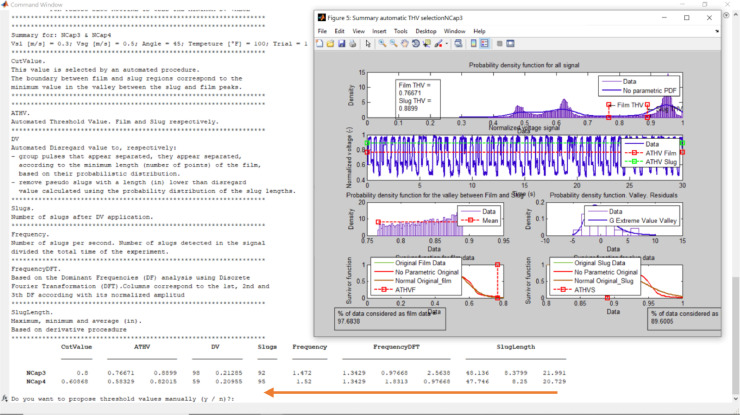
Changing the proposed threshold values.

After that, the algorithm asks the film and slug threshold values for the pair of capacitance sensors, plots the results, and presents a summary. The run stops waiting for the user response (Fig. 13): Do you want to propose threshold values manually (y / n)? The user can make a decision based on the values reported in the summary table and in the several plots, which are displayed.

**Fig. 14:**
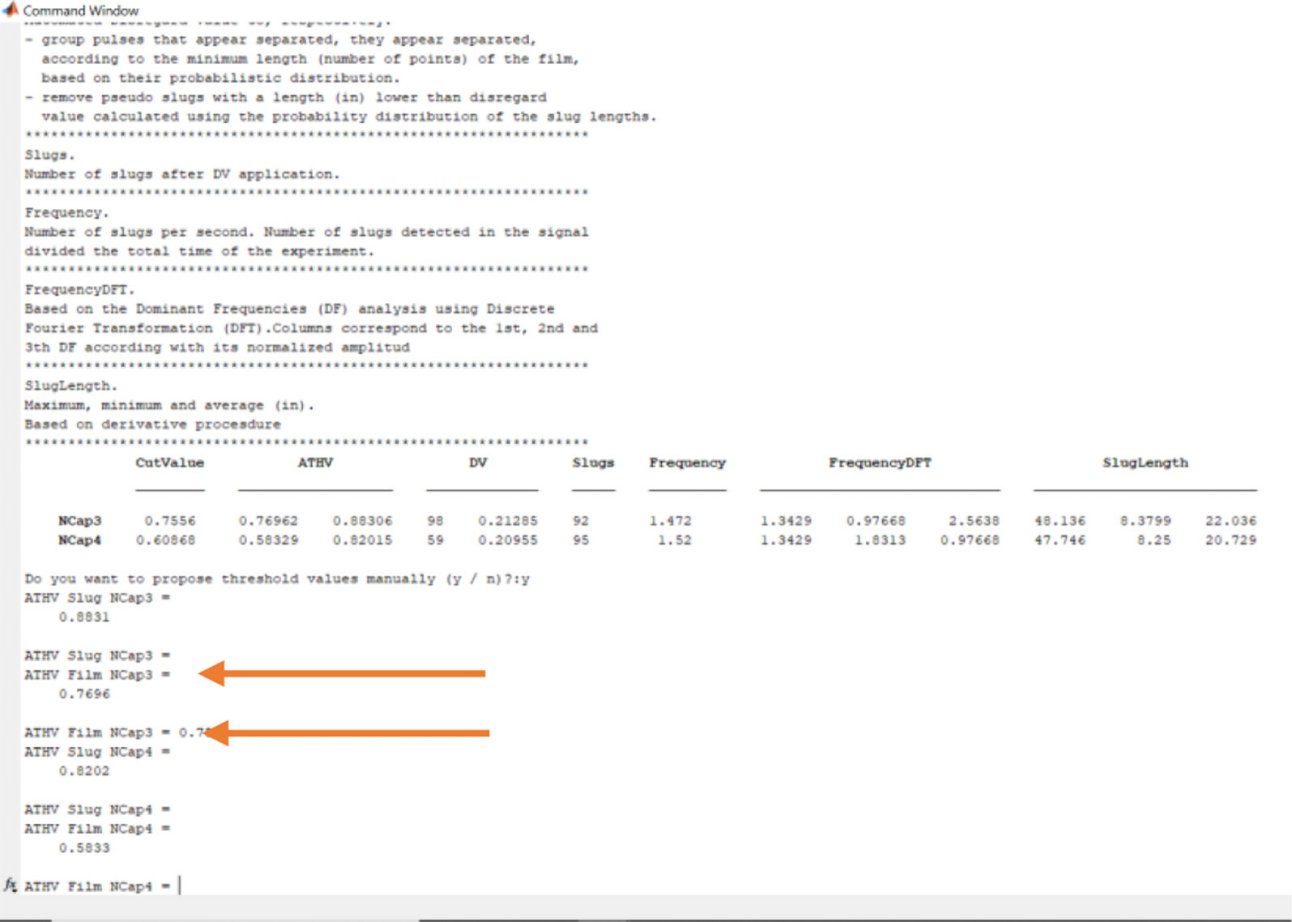
The film threshold value for capacitance sensor 3, changed from 0.7696 to 0.75.

Write y + Enter. The code will remember the actual threshold values (slug and film) and wait for user response. Just click Enter to keep the value or write down the new value (Fig. 14). The code will recalculate and show the results. This iterative process finishes when the user responds n to the question. Do you want to propose threshold values manually (y / n)? The user must answer this question for each capacitance sensor, in each station, and each experiment.

### Example 3-Reviewing the disregard values

Based on Example 2, consider that the user wants to check the disregard values for the group subroutine and void pseudo-slug subroutine [1]. Then, in the *Options* sheet, cells A2-A6 are equal to 1.

Make sure thatExample 3.xlsxis closed. Run BSignalProcessing2020.m. The code asks for the path of the data file folder. From the file explorer, copy and paste the path and press Enter. A list of the xls files existing in the folder will be displayed. Select Option 3 and press Enter.

**Fig. 15:**
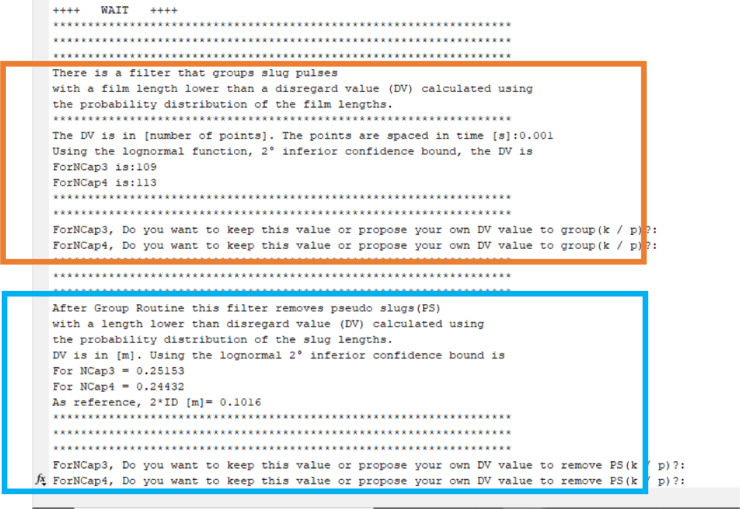
Selecting your own cut value.

After the film-slug boundary selection, the code calculates the disregard values (DV) for the grouping routine (orange rectangle in Fig. 15). The DVs are presented as a “number of points” equally spaced in time. In this example, the acquiring time step is 0.001 s. In this interactive mode, the user can change the proposed DV value. Then, the pseudo slug routine starts (blue rectangle in Fig. 15). In this case, the DV is reported in meters.

## Declaration of Competing Interest

The authors declare that they have no known competing financial interests or personal relationships that could have appeared to influence the work reported in this paper.

## Appendix Supplementary materials

Image, application 1

Image, application 2

Image, application 3

Image, application 4

Image, application 5
